# Effect of Salt Stress on Growth, Na^+^ Accumulation and Proline Metabolism in Potato (*Solanum tuberosum*) Cultivars

**DOI:** 10.1371/journal.pone.0060183

**Published:** 2013-03-22

**Authors:** Rinse Jaarsma, Rozemarijn S. M. de Vries, Albertus H. de Boer

**Affiliations:** Department of Structural Biology, Faculty of Earth and Life Sciences, Vrije Universiteit, Amsterdam, The Netherlands; University of Massachusetts Amherst, United States of America

## Abstract

Potato (*Solanum tuberosum*) is a major crop world-wide and the productivity of currently used cultivars is strongly reduced at high soil salt levels. We compared the response of six potato cultivars to increased root NaCl concentrations. Cuttings were grown hydroponically and treated with 0 mM, 60 mM and 180 mM NaCl for one week. Growth reduction on salt was strongest for the cultivars Mozart and Mona Lisa with a severe senescence response at 180 mM NaCl and Mozart barely survived the treatment. The cultivars Desiree and Russett Burbank were more tolerant showing no senescence after salt treatment. A clear difference in Na^+^ homeostasis was observed between sensitive and tolerant cultivars. The salt sensitive cultivar Mozart combined low Na^+^ levels in root and stem with the highest leaf Na^+^ concentration of all cultivars, resulting in a high Na^+^ shoot distribution index (SDI) for Mozart as compared to Desiree. Overall, a positive correlation between salt tolerance and stem Na^+^ accumulation was found and the SDI for Na^+^ points to a role of stem Na^+^ accumulation in tolerance. In stem tissue, Mozart accumulated more H_2_O_2_ and less proline compared to the tolerant cultivars. Analysis of the expression of proline biosynthesis genes in Mozart and Desiree showed a clear reduction in proline dehydrogenase (*PDH*) expression in both cultivars and an increase in pyrroline-5-carboxylate synthetase 1 (*P5CS1*) gene expression in Desiree, but not in Mozart. Taken together, current day commercial cultivars show promising differences in salt tolerance and the results suggest that mechanisms of tolerance reside in the capacity of Na^+^ accumulation in stem tissue, resulting in reduced Na^+^ transport to the leaves.

## Introduction

Accumulation of salts in soil has a negative effect on the production of a wide variety of crops. For various reasons the area of salt affected soils will rapidly expand in the near future. As the world population continues to grow, the availability of renewable freshwater resources for agriculture will decrease, and simultaneously the area of irrigated land will increase in the attempt to satisfy the need for more food. In view of the wealth of salt stress related studies on model plants and crops, there are relatively few studies that describe which traits remained in current potato cultivars that might be a starting point to improve tolerance of potato to salt. Potato is the fourth most cultivated crop world-wide and in (semi)arid areas where salt stress is a serious problem, productivity is considerably reduced. Wild potatoes growing under harsh conditions in the Andes are relatively stress tolerant, but extensive breeding and selection for traits other than abiotic stress tolerance have resulted in cultivars that are considered moderately salt tolerant [1] (FAO, 2010).

Salt stress results in a reduction in biomass production, a decrease in shoot length, induction of senescence response or earlier plant death. Studies over the last years have revealed a number of important strategies to improve salt tolerance. One strategy is the controlled influx of Na^+^ into the root cells. A comparison of Na^+^-influx in root cells of the glycophyte *Arabidopsis thaliana* and those of the halophyte *Thellungiella halophila* shows that ion channels in the halophyte species are much more selective for Na^+^ than those of *Arabidopsis* [2]. In general, Na^+^-influx is mediated by non-selective cation channels (NSCCs) [3], but it is unclear which class of NSCC is most important for Na^+^-influx nor the underlying genes have been identified [4]. Excess Na^+^ ions that reach the transpiration stream in the root system are destined for the shoot by transport through the xylem. However, plants have the ability to absorb Na^+^ from the xylem sap to surrounding tissue by means of Na^+^-transporters that belong to the HKT family [5]. This mechanism seems to be particularly important for glycophytes that cannot tolerate high Na^+^ levels in the leaves, like *Arabidopsis thaliana* columbia ecotype. *Arabidopsis* ecotypes from coastal areas or saline soils have a weak allele of the *AtHKT1;1* gene, have elevated leaf Na^+^ levels and are more salt tolerant than *Arabidopsis thaliana* Columbia ecotype [6], [7]. This is probably due to a higher capacity of these coastal ecotypes to sequester Na^+^ in the leaf vacuoles. Plants well adapted to salt like halophytes, have a high capacity to accumulate Na^+^ ions in the large central vacuole [8]. Vacuolar sequestration of Na^+^ avoids toxic levels of Na^+^ in the cytosol and reduces at the same time the water potential of a large volume of the cell. Sequestering Na^+^ ions into vacuoles has a large impact on the cellular osmotic potential. To balance the water potential of the cytosol with the apoplast and vacuolar lumen, plants produce osmotically active solutes like proline in response to salt stress [9]. Besides regulation of osmotic pressure, proline has been shown to stabilize proteins and membranes, protect plants against free radical-induced damage and proline maintains appropriate NADP^+^/NADPH ratios [10], [11]. A key mechanism in proline metabolism is the reciprocal regulation of the proline biosynthesis gene *P5CS1* and the proline degradation gene *PDH* [9], [12]. During salt stress, *P5CS1* is induced and *PDH* is repressed [12]–[14]. Over-expression of the *P5CS* gene from *Arabidopsi*s in potato strongly stimulated proline production particularly in the presence of 100 mM NaCl and improved salinity tolerance with respect to tuber yield and weight [15].

Once Na^+^ is inside the cytosol of plant cells and is not sequestered in the vacuole, it may interfere with the function of (K^+^) as a co-factor for a range of enzymes, since Na^+^ replaces K^+^ physically but not functionally [16]. The ability to maintain K^+^ homeostasis during salt stress is considered a characteristic of more salt tolerant plants [16], [17]. One important novel determinant for salt tolerance is the ability to retain K^+^ in the root upon exposure to NaCl as was shown for a range of barley and wheat cultivars by using the vibrating probe technique [18], [19].

Besides disturbing K^+^ homeostasis, enhanced cellular Na^+^ levels induce the production of reactive oxygen species (ROS), such as superoxide radicals (•O^2−^), hydrogen peroxide (H_2_O_2_), and hydroxyl radicals (•OH) [20]. A comparison of H_2_O_2_ accumulation in two rice cultivars showed that H_2_O_2_ accumulation is much higher in the salt sensitive cultivar [21]. ROS can damage cellular structures and plants have to a variable extent ROS scavenging antioxidant metabolites and enzymes. Potato cultivars containing elevated levels of antioxidant enzymes were found to be more salt tolerant [22].

In this study, we have investigated the salinity tolerance of six potato cultivars by analyzing physiological parameters, Na^+^ and K^+^ contents, H_2_O_2_ contents and proline contents in roots, stems and leaves. In leaf and stem tissues expression of genes involved in the proline pathway was analyzed. We related growth performance to Na^+^ accumulation in the different tissues and we analyzed differences in distribution of Na^+^ in aerial parts of all cultivars to explore differentiations between cultivars in the movement of Na^+^ through the plant. A comparison of the responses of these cultivars to salt stress will be useful in identifying the mechanisms of salt tolerance in potato. Important differences were found in the accumulation of Na^+^ ions and proline in the shoot. These differences can be used as a starting point to elucidate the molecular mechanisms that enable potato plants to cope with salt stress.

## Results

### Salt stress induced fresh weight reduction

**Figure 1:**
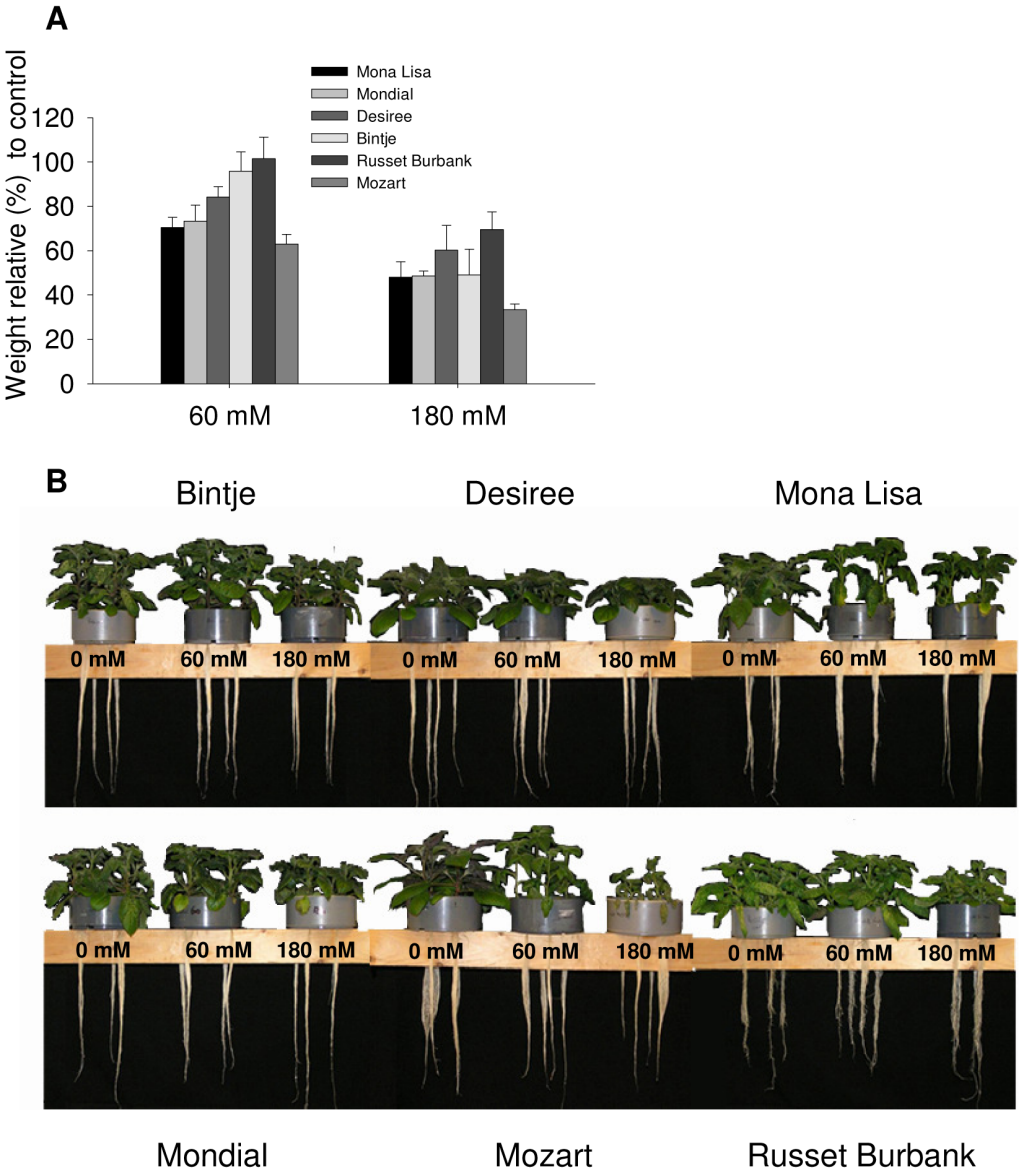
Effect of 60 mM and 180 mM NaCl on the growth of six potato cultivars. A. Diversity in the salt tolerance of 6 cultivars, shown as decreases in fresh weight after growth on hydroponic culture, relative to plant growth in the absence of NaCl. Results are means of three replicates and expressed as percentages from the weight of control plants. (±S.E.). The average coefficient of variation (CV) for all cultivars grown at 0 mM NaCl was 0.3 (0.2 to 0.4), for cultivars grown at 60 mM the CV was 0.4 (0.2 to 0.5) and for cultivars grown at 180 mM the CV was 0.5 (0.3 to 0.7). B. Representative photographs of the six potato cultivars grown on hydroponics for three weeks after treatment with either 0, 60 or 180 mM NaCl during the last seven days.

Based on the outcome of a pilot experiment with 10 cultivars grown as cuttings in agar medium with NaCl, tolerant and sensitive cultivars were selected for this study. These were the cultivars Desiree, Russet Burbank, Bintje, Mondial, Mona Lisa and Mozart. To evaluate the salinity tolerance of these six cultivars, plants were propagated as cuttings for two weeks and then grown on hydroponics (1/2 strength Hoagland) for three weeks, whereat during the last week of the experiment plants were exposed to three levels of salt (0 mM, 60 mM and 180 mM NaCl). Fresh weight production and a number of salt related cellular parameters were measured in response to these salt treatments. The cultivars Desiree, Russet Burbank and Bintje did not show a significant reduction in fresh weight (FW) when exposed to 60 mM NaCl as compared to growth at 0 mM NaCl (Figure 1A). The FW production of the other three cultivars decreased after 60 mM NaCl compared to the 0 mM NaCl treatment. Two-way ANOVA showed a significant varietal effect of salinity on the fresh weight of the six potato cultivars after the 60 mM NaCl treatment. At 60 mM NaCl Mozart showed the strongest growth reduction: around 40%. At 180 mM, all cultivars showed a significant reduction in FW over the 7 d period; however, no significant difference in growth reduction was found at 180 mM NaCl between cultivars. The lack of difference in growth reduction between cultivars indicates that the 180 mM NaCl treatment is relatively severe for potato plants to cope with. The cultivars Mozart and Mona Lisa showed a severe senescence response at 60 mM and 180 mM NaCl (Figure 1B) and at 180 mM Mozart barely survived. The cultivar Desiree did not show any senescence response at elevated NaCl levels, including 180 mM NaCl.

### Effect of salt stress on tissue levels of Na^+^

**Figure 2:**
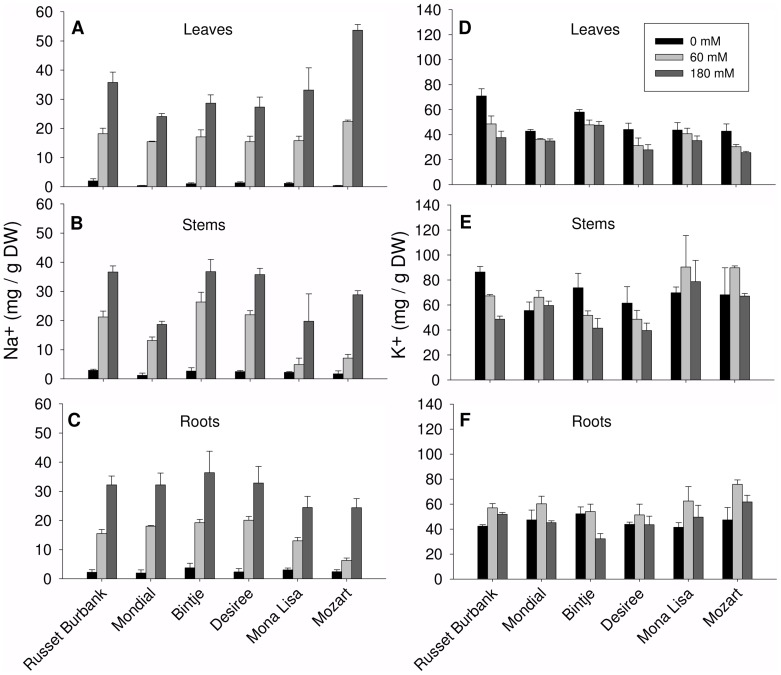
Sodium and potassium concentrations in the leaves, stems and roots of the six cultivars. Plants were grown hydroponically for three weeks and treated with either 0, 60 or 180 mM NaCl during the last seven days. At harvest, leaves, stems and roots were collected separately. Roots were rinsed with deionized water before analysis. Tissues were dried overnight at 70°C and Na^+^ and K^+^ contents in the tissue extracts were determined by using High Pressure Liquid Chromotography. Four plants in each treatment were used. The experiment was repeated three times and the results show the mean±SE.

The amount of Na^+^ and K^+^ accumulated in leaf, stem and root tissue is shown in Figure 2. A two way ANOVA showed a significant varietal effect of salinity on the Na^+^ concentrations in the leaf and stem tissues but not in the root tissues. The absence of a significant varietal effect in root tissues is caused by the high variation in Na^+^ concentrations in roots grown at 180 mM NaCl. A significant varietal effect of salinity on roots was found when values from plants grown at 180 mM NaCl were excluded from the statistical analysis, again indicating the 180 mM NaCl treatment is rather severe for all potato cultivars. Mozart and Mona Lisa, cultured at 60 mM NaCl, accumulate significantly less Na^+^ in the roots and stems as compared to the other cultivars. In Mozart, these relatively low Na^+^ levels in root and stem are combined with the highest leaf Na^+^ concentration of all cultivars. This results in a much lower Na^+^ root:leaf ratio for Mozart (around 0.25) as compared to Desiree (1.25) at 60 mM NaCl.

### Effect of salt stress on tissue levels of K^+^

No significant differences were observed in K^+^ concentrations between cultivars in leaves, stems or roots after salt treatment (Figure 2). In leaf tissue the overall trend is a reduction in K^+^ with increasing salt stress and in roots it is remarkable that at 60 mM NaCl the K^+^ concentrations are higher than in roots grown without salt. Interestingly, the salt sensitive cultivars Mozart and Mona Lisa maintained K^+^ levels in stem tissue at control level after 60 mM and 180 mM salt treatment (Figure 2D); this despite the fact that Na^+^ levels drastically increased at 180 mM NaCl (Figure 2B). We calculated the leaf K:Na ratio as shown in Table 1. Five cultivars show a comparable (no significant differences) K:Na ratio ranging from 2.1 to 2.8 at 60 mM NaCl and 1.1 to 1.7 after 180 mM NaCl treatment. However, Mozart is the exception and a multiple comparison analysis shows that Mozart has a significantly lower K:Na ratio as compared to the other cultivars in leaves.

**Table 1:** K^+^:Na^+^ ratio calculated for leaf tissue of the six cultivar grown hydroponically for three weeks and treated with 60 or 180 mM NaCl.

mM NaCl	Russet Burbank	Mondial	Bintje	Desiree	Mona Lisa	Mozart
60	2.8±0.6	2.4±0.04	2.9±0.3	2.1±0.7	2.7±0.5	1.4±0.05
180	1.1±0.3	1.5±0.1	1.7±0.3	1.1±0.3	1.2±0.4	0.5±0.03

Results are the mean±SD for three biological replicates

### Plant Tolerant Index and Shoot Distribution Index of potato cultivars

**Figure 3:**
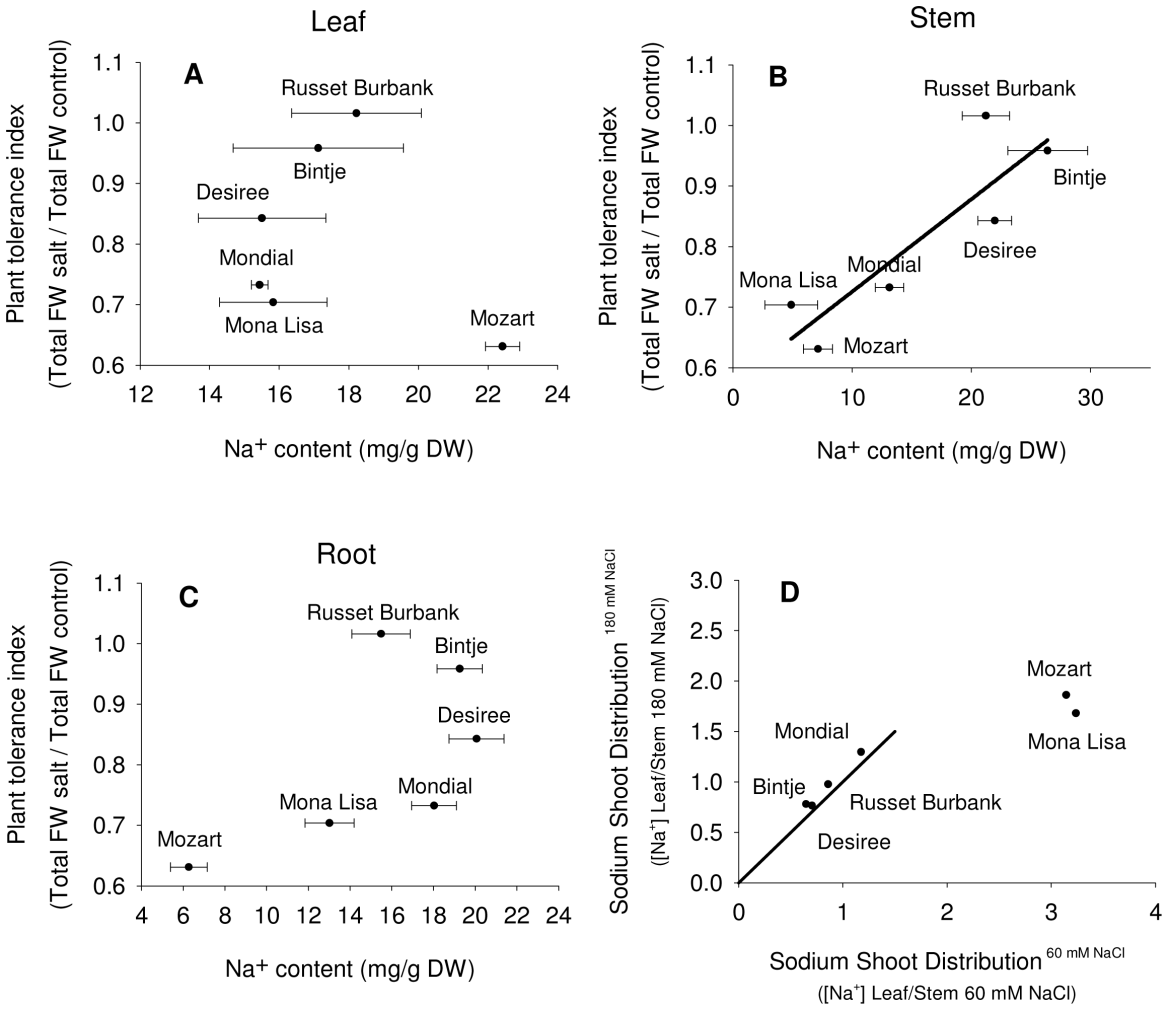
Plant tolerance index related to salinity tolerance and the sodium shoot distribution. Relationship between (A) leaf, (B) stem and (C) root Na^+^ content and plant salinity tolerance, as measured by total fresh weight (FW) in salt stressed plants÷total FW in control plants (modified from Jha et al., 2010), in six potato cultivars. Results are the mean ± SE of three biological replicates. (D) Relationship (Shoot Distribution Index; SDI) between sodium concentrations in leaves and in stems, as determined by (sodium concentrations in leaves)÷(sodium concentrations in stems) for plants treated with 60 mM NaCl (x-axis) and plants treated with 180 mM NaCl (y-axis). The inserted dotted line represents the function y = (f)x. In both salt treatments, four cultivars have an SSD value of around one, what indicates that these four cultivars distribute sodium equally between leaves and stems. The SDI of Mozart and Mona Lisa reaches values of more than three after 60 mM NaCl treatment, what implies that sodium concentrations in the leaves are three times higher as compared to stems, after the 60 mM NaCl treatment.

For plants exposed to 60 mM NaCl we calculated the plant tolerance index (PTI) as FW^salt^/FW^control^ and related this to the amount of Na^+^ in leaves, stems and roots of all cultivars (Figure 3A, B and C). For stem tissue, but not for leaf- and root tissue, this analysis shows a positive correlation for all six cultivars: Mozart and Mona Lisa combine low stem Na^+^ accumulation with a low PTI, and Russet Burbank and Bintje accumulate significantly higher concentrations of stem Na^+^ while maintaining normal levels of growth (high PTI) in 60 mM NaCl (Figure 3B).

As an index for the distribution of sodium between stem and leaf tissues we calculated the Shoot Distribution Index (SDI) as [Na^+^]^leaves^/[Na^+^]^stems^ for 60 mM and 180 mM NaCl treatments and plotted SDI^60^ against SDI^180^ (Figure 3D). The SDI shows an interesting difference between the two sensitive cultivars (Mozart and Mona Lisa) and the other four cultivars. First, the more tolerant cultivars have an SDI of around 1 and this remains constant at both NaCl concentrations (Figure 3D). In contrast, Mozart and Mona Lisa have an SDI of around 3 at 60 mM NaCl and the SDI decreases when grown at 180 mM NaCl.

### H_2_O_2_ concentration of potato cultivars

**Figure 4:**
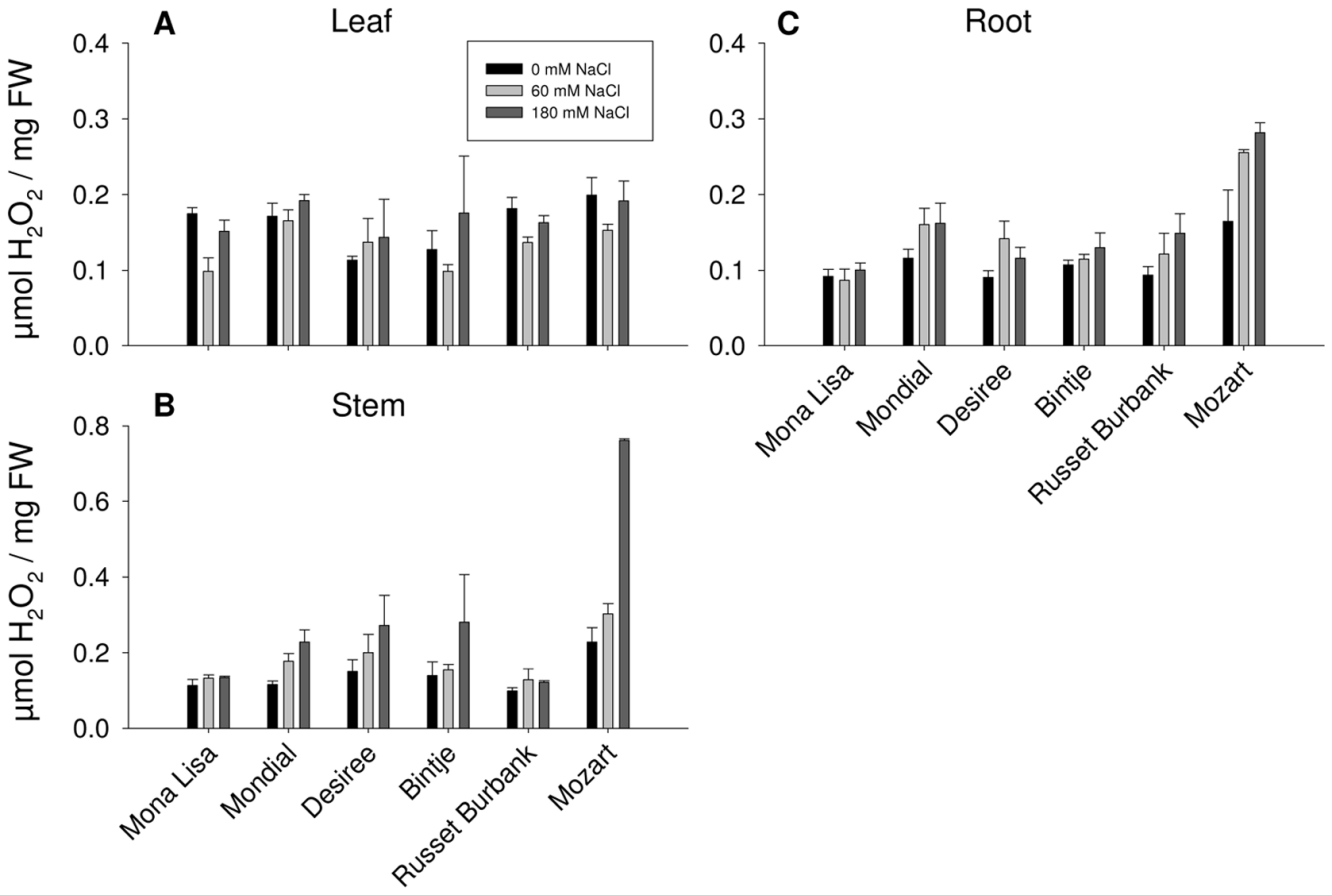
The effect of NaCl treatments on hydrogen peroxide concentrations in (A) leaves, (B) stems and (C) roots of six potato cultivars. Data represents the means±SE for three biological replicates.

To investigate the effect of salt stress on net ROS accumulation in the cultivars, we measured the H_2_O_2_ concentrations in leaves, stem and roots of control and salt treated plants. In leaves, a two way ANOVA showed no varietal effect of the salt treatment, although the statistical analysis did show that the salinity treatment had effect on the H_2_O_2_ accumulation in all cultivars (Figure 4A). Notably, plants grown at 60 mM NaCl have lower H_2_O_2_ concentrations in leaves than control plants. In stem tissue H_2_O_2_ levels differed between cultivars after the salt treatment, with Mozart having significantly higher H_2_O_2_ levels as compared to the other cultivars (Figure 4B). The very high concentrations of H_2_O_2_ in the stem of Mozart plants grown at 180 mM salt correspond with the poor growth performance of these plants (Figure 1). Although a two way ANOVA did not show a varietal effect in root tissue, a trend was found that Mozart has higher levels of H_2_O_2_ after salinization in root tissue compared to other cultivars (Figure 4C). For the stem tissue and for the root tissue a multiple comparison analysis showed that Mozart has increased concentrations of H_2_O_2_ during salinity.

### Proline concentration of potato cultivars

**Figure 5:**
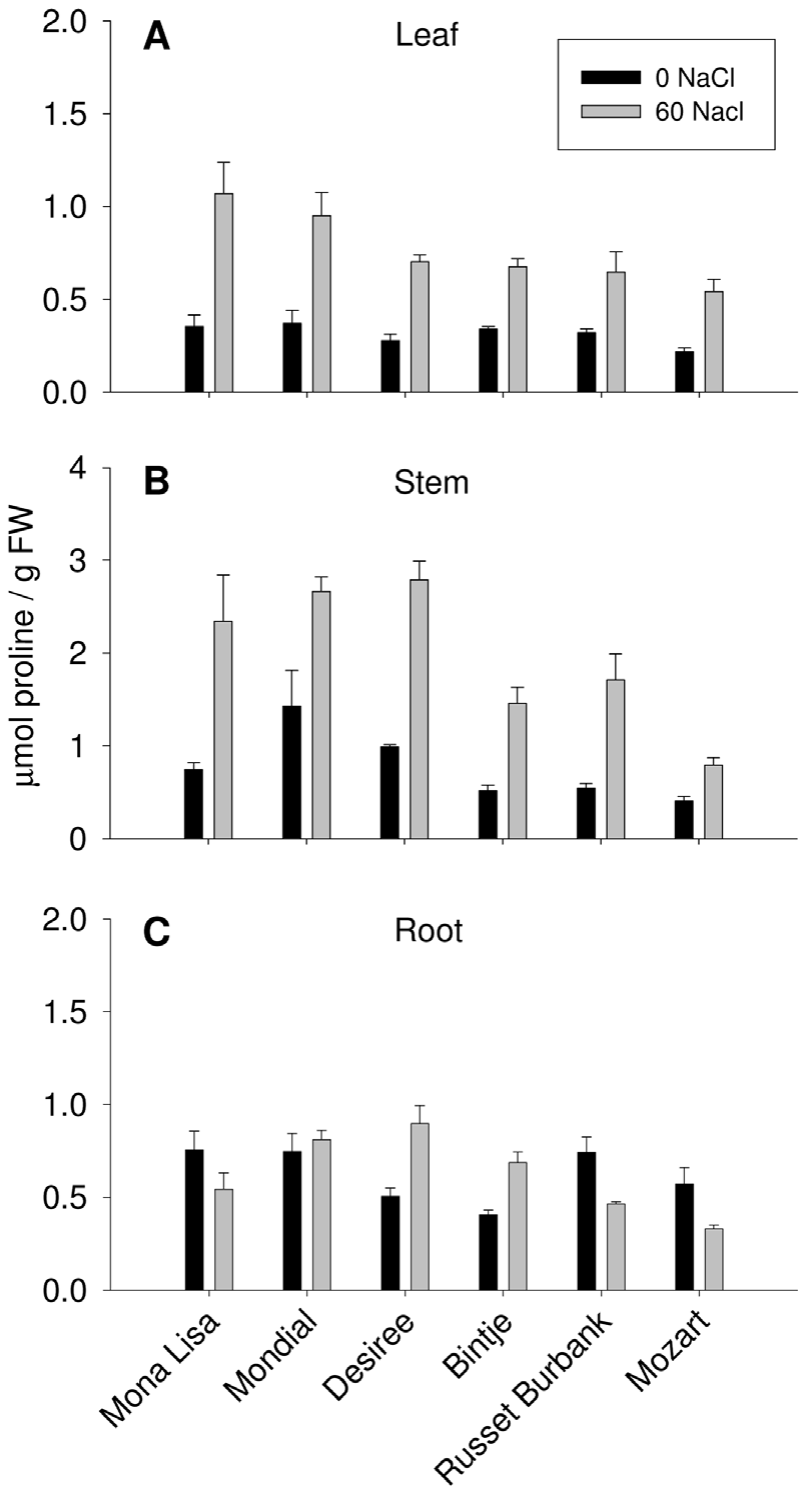
The effect of NaCl treatments on proline concentrations in (A)leaves, (B) stems and (C) roots of six potato cultivars. Data represents the means±SE for three biological replicates.

We analyzed the proline content of leaf-, stem- and root tissues of plants grown at 0 and 60 mM NaCl (Figure 5). In leaf and stem tissue, a two-way ANOVA indicates a significant effect of salinity on the proline content (Table 2). Upon salt stress, the proline concentrations increased in leaf- and stem tissues for each cultivar (Figure 5). In stem tissue, Mozart accumulates significantly less proline as compared to Desiree and Mondial after the 60 mM NaCl treatment. In root tissue, a two way ANOVA analysis showed a varietal effect on the proline content of enhanced salinity (Table 2). After salt treatment, proline concentrations in roots remained unchanged in the cultivars Mona Lisa and Mondial, increased in the cultivars Desiree and Bintje and decreased in the cultivars Russet Burbank and Mozart (Figure 5C).

**Table 2:** Results of two way ANOVA of cultivar (C) and salinity (S) effects and their interaction (C×S) for the proline concentrations in leaf, stem and root tissues.

Dependent variable	Independent variable		
Proline	C	S	C×S
Leaf tissue	1.6	32.7 **	0.7
Stem tissue	4.2	28.8 **	0.8
Root tisue	1.8	0.1	2.8*

Numbers represent F values at 5% level, * p<0.05, ** p<0.001

### Expression profiling of enzymes involved in the proline pathway

**Figure 6:**
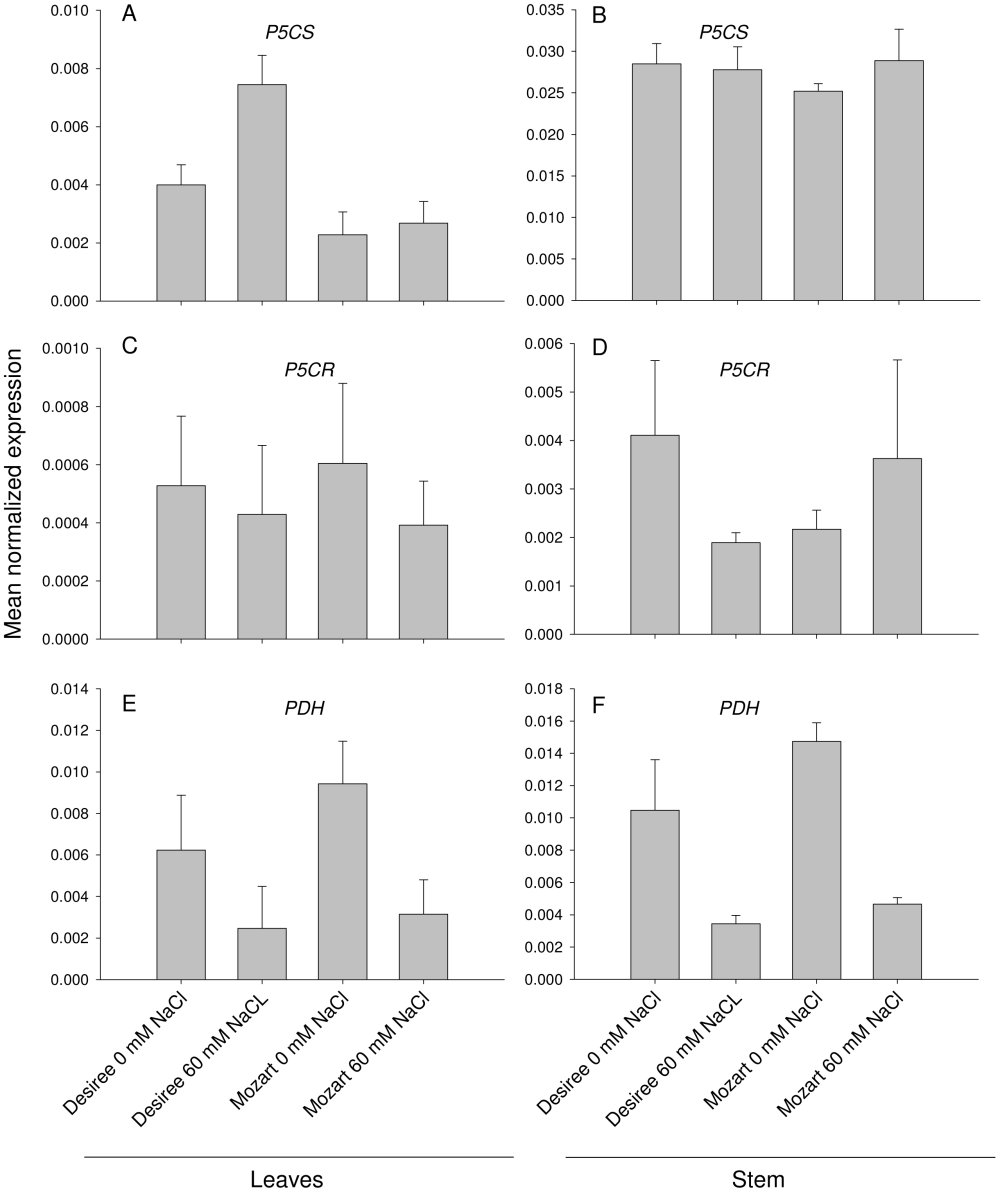
Expression analysis of genes involved in the proline metabolism pathway. The effect of NaCl treatments (0 and 60 mM) on the expression profile of *P5CS1*, *P5CR*, *PDH*, in the leaves (A, C, E) and the stems (B, D, F) of the cultivar Desiree and the cultivar Mozart. Results are the mean±SE for three biological replicates.

Potato expressed sequence tags (EST's) were found for genes encoding enzymes involved in the proline pathway by homology search using genes from *Arabidopsis* as described in Material and Methods. Two cultivars contrasting in the properties analysed so far, Mozart versus Desiree, were chosen for expression analysis of three genes related to proline biosynthesis (*P5CS1* and *P5CR*) and breakdown (*PDH*). Expression profiles were determined in leaf and stem tissue of control and 60 mM NaCl treated plants (Figure 6). Transcript levels of both *P5CS1* and *P5CR* were much higher in stem than in leaf tissue and stem expression is constitutively high for both cultivars (Figure 6A, B, C, D). In leaves, salinity induced a two-fold increase in *P5CS1* expression of Desiree, whereas Mozart *P5CS1* did not respond (Figure 6A). A clear salt response is shown by the *PDH* gene in both cultivars: both in stem and leaf tissue salt stress reduced the expression levels 3- to 4-fold (Figure 6E, F).

## Discussion

Despite the agricultural importance of potato little is known about the differences and mechanisms of salt tolerance in currently used potato cultivars. A few studies have compared potato cultivars or addressed known molecular mechanism of salt tolerance like ion homeostasis, proline accumulation, activity of antioxidant enzymes and vacuolar sequestration [22]–[25]. The ability to exclude sodium from the shoot is an important determinant of salt tolerance in both monocots and dicots [26]–[29]. However, the salt sensitive *Arabidopsis* ecotype Columbia grown at moderate salt concentrations keeps the shoot Na^+^ concentration much lower than do coastal, more salt tolerant, ecotypes [30]. This strategy has a limited capacity since growth of *Arabidopsis* ecotype Colombia at high salt is severely limited due to a strong increase in shoot Na^+^ concentration. Like *Arabidopsis*, potato plants are dicots and shoots from potato plants are easily separable enabling to distinguish ion homeostasis between leaf- and stem tissue. The more salt sensitive potato cultivars of this study, Mozart, Mona Lisa and Mondial keep the average stem Na^+^ concentration lower than the more salt tolerant cultivars Russet Burbank and Desiree (Figure 2 and 3). The more salt tolerant potato cultivars seem to have a similar strategy as the coastal *Arabidopsis* ecotypes when grown at intermediate NaCl level. The positive relationship found in this study between the PTI and stem Na^+^ concentrations is partly comparable to what was recently reported in a study with a range of *Arabidopsis* ecotypes [7]. Jha and co-workers suggested that *Arabidopsis* may use mechanisms involved with Na^+^ tissue tolerance, such as intracellular compartmentation and increased accumulation of compatible solutes, as was hypothesized before [28] and apparently, some of the potato cultivars respond in this way. In addition, a major role for stem tissue can be assigned in Na^+^ tissue tolerance in potato cultivars. Another striking difference between Mozart and Mona Lisa compared to the more salt tolerant cultivars is the way they distribute the Na^+^ between the stem and the leaves. The more tolerant cultivars have an SDI of 0.75 to 1.5, indicating that the tolerant cultivars distribute Na^+^ equally between stems and leaves. Mozart and Mona Lisa have a SDI of 3 (Figure 3D) indicating that the sensitive cultivars transport relatively more Na^+^ to leaves. When grown at 180 mM NaCl, the more tolerant cultivars keep this ratio the same as at 60 mM NaCl. It seems that salinity tolerance in potato is related to a tissue tolerance mechanism, with those cultivars accumulating more Na^+^ in stem tissue are more tolerant and it will be of interest to see whether differences in *HKT1* expression in the stems correlate with this SDI [5], [31]. The lack of senescence in the more tolerant cultivars during elevated leaf Na^+^ levels (Figure 1) suggests that tolerant cultivars detoxify elevated leaf Na^+^ levels by intracellular compartmentation. Intracellular compartmentation might point to the importance of a high capacity for Na^+^-sequestration in the vacuoles as has been shown before [28], [32]. Using a tissue culture technique (cv. Desiree), Queiros et al. isolated tonoplast-enriched vesicles from non-adapted and 150 mM NaCl-tolerant suspension-cultured cells exposed to salt stress [25]. The activity of the vacuolar pumps and the Na^+^/H^+^-antiport were higher in tonoplast vesicles from the salt-tolerant line. In view of the growth sensitivity and senescence response of Mozart at leaf Na^+^ concentrations higher than those of the tolerant cultivars, we hypothesize that the vacuolar sequestration capacity of Mozart is lower than that of the more tolerant cultivars (Jaarsma and de Boer, unpublished data).

The strong senescence response observed for Mozart when grown at 60 mM and 180 mM NaCl correlates well with the higher H_2_O_2_ concentrations measured in the different tissues. Notably, stem tissue of plants grown at 180 mM NaCl had high levels of H_2_O_2_. This result is in line with the report of Aghaei et al. [23] namely that in the salt tolerant potato cultivar Kennebec activities of ROS scavenging enzymes like ascorbate peroxidase, catalase and glutathione reductase were increased by salt stress, whereas the corresponding activities in the salt sensitive cultivar Concord were decreased [23].

The potato cultivars respond to 60 mM salt stress with an increase in proline concentration, with the highest accumulation in the stem tissue; proline and Na^+^ concentrations in the stem of the different cultivars correlate reasonably well. However, cultivars having the highest PTI (Russet Burbank, Bintje) do not necessarily accumulate more proline than cultivars having a lower PTI, like Mondial (Figure 5). Although it is generally agreed upon that proline accumulation is important for plants to tolerate environmental stresses, a good correlation between proline accumulation and stress tolerance is not always obvious [24], [33], [34]. In proline synthesis P5CS and P5CR are the main enzymes, and in proline catabolism PDH has a key function [9]. Proline catabolism seems to be an important mechanism to regulate proline levels; e.g. in the halophyte *Thellungiella*, *PDH* expression is much lower than in the glycophyte *Arabidopsis*, what correlates with high proline concentrations in *Thellungiella* [35]. Also here we observed that both in stem and leaf tissue expression of the potato *PDH* gene is strongly reduced upon salt treatment in both cultivars tested. A differential response between the cultivars was only observed for the *P5CS* gene in leaf tissue, with a two-fold higher expression in Desiree leaves and no change in Mozart upon salt stress. Although the salt induced increase in proline concentrations is explained by the reduced *PDH* expression in Desiree and Mozart and the increase in *P5CS* expression in Desiree, the 3-fold higher proline levels in the stem of Desiree as compared to Mozart do not correlate with the differences in gene expression.

The ability to maintain K^+^ homeostasis during salt stress is considered a characteristic of more salt tolerant plants [16], [17]. A comparison of a salt tolerant (Kennebec) and salt sensitive (Concord) cultivar [23] and a comparison of four other potato cultivars [24] showed that K^+^ levels decreased, even when plants were moderately salt stressed (50–75 mM NaCl). In line with these studies, K^+^ levels decreased in leaf tissues of the cultivars tested in this study. However, it is noteworthy that we did not observe striking differences in K^+^ contents between cultivars in any tissue, despite the strong accumulation of Na^+^ (Figure 2).

In conclusion, the commercial potato cultivars tested in this study show considerable variation in sensitivity to salt stress, as evidenced from the growth performance. At moderate salt treatment (60 mM NaCl) PTI shows a positive correlation with the stem Na^+^ accumulation. A clear distinction between the sensitive and more tolerant cultivars is the capacity of the tolerant cultivars to accumulate Na^+^ ions from the transpiration stream into their stem tissue. In addition, the salt sensitive cultivars transport relatively more Na^+^ to leaf tissue as compared to the salt tolerant cultivars.

## Materials and Methods

### Plant material and growth conditions

Two-week old cuttings of six potato (*Solanum tuberosum*) cultivars Desiree, Mona Lisa, Bintje, Mondial, Mozart and Russet Burbank were planted on four litre pots containing ½ strength Hoagland solution. Each pot contained four cuttings of the same cultivar surrounded by a rockwool plug embedded in styrofoam on top of the pot. Plants were maintained in a growth chamber under a 16∶8 light:dark photoperiod, 15°C∶ 24°C night:day temperature and 70% humidity. After two weeks, plants were subjected to three salt treatments, 0 mM NaCl, 60 mM NaCl and gradually to 180 mM NaCl in steps of 60 mM NaCl every two days to prevent osmotic stress. All salt treatments were performed in triplicate. After seven days of salt treatment, the plants were harvested, measured and analyzed. Total fresh weight (FW) was immediately determined after harvest and averaged per pot. Each pot was considered as one measurement. To standardize the data, the results were expressed as the relative reduction of total FW in comparison to the control treatment.

### Chemical analysis

After weighting, fresh plant material was divided into two parts. One part was frozen in liquid nitrogen and stored at −80°C for later use. The remaining fresh plant material was dried at 70°C for 24 hours. For determination of Na^+^ and K^+^ concentrations, 100 mg dried plant material was extracted by one hour boiling in 5 ml MilliQ. The solution was filtered through 0.2 µm filters (Whatman, England) and Na^+^ and K^+^ contents in the filtrate were analyzed using high-performance liquid chromatography (HPLC, Shimadzu Japan). The HPLC system was equipped with a ø 4.6 mm×125 mm Shodex IC YS-50 column (Showa Denko). As an eluent, 4.0 mM methane sulfonic acid was used in HPLC graded H_2_O (J.T. Baker, The Netherlands) with a flow rate of 1 ml/min. Final ion concentrations in the filtrate were calculated according to a calibration curve.

Proline content was determined according to Bates et al. (1973) and extracted from 30 mg dried plant material of root, stem or leaf tissue with 2 ml 3% Sulphosalicyl acid. After 10 minutes of centrifuging at 17.500 *g*, 500 µl supernatant was transferred to a mixture containing 500 µl glacial acetic acid and 500 µl ninhydrine reagent. The ninhydrine reagent contains glacial acetic acid and orthophosphoric acid (6 M) (3∶2, v/v) and 25 mg ninhydrine per ml ninhydrine reagent. After incubating for one hour at 100°C the tubes were cooled and 1 ml toluene was added and mixed. The absorbance of the upper phase was spectrophotometrically determined at 520 nm after ten minutes. The proline concentration was calculated according to a calibration curve. Hydrogen peroxide was measured with the Amplex Red Peroxide Assay Kit, A22188 according to the manufacturer's instructions (Invitrogen).

### Statistical analysis

The pot experiment was arranged in a randomized design with three salt treatments. Per cultivar each salt treatment was done in triplicate. One pot contained four plants that were pooled at the end of the experiment and subsequently considered as one measurement. Analysis of variance (ANOVA) and multiple comparison test (Tukeys test) was computed using SPSS computer package. Differences between treatments were considered significant only when p<0.05 after statistical analysis. The coefficient of variation (CV) was calculated per cultivar for each salt treatment. Subsequently, CV per cultivar for each salt treatment was averaged per salt treatment of all six cultivars.

### Gene expression analysis

RNA was isolated from leaves and stems using the Nucleospin RNeasy plant mini kit (Qiagen, Leusden, The Netherlands) including DNase treatment. Tissue was ground in liquid nitrogen using a pestle and mortar. Approximately 100 mg of ground tissue was used for RNA isolation according to the manufacturer's instructions. For real-time qRT-PCR, cDNA was synthesized from 1–4 µg RNA using Superscript II (invitrogen) in a 20 µl reaction volume containing 5 µM oligo dT (T15) primer according to the manufacturer's protocol. The potato elongation factor *ef1α* gene (AB061263, forward primer 5′-3′ ATTGGAAACGGATATGCTCCA, reverse primer 5′-3′ TCCTTACCTGAACGCCTGTCA.) was used as a reference gene since this gene was proven to be stable during salt stress [36]. Quantitative RT-PCR (QRT-PCR) reactions contained 100 ng cDNA, 0.5 pmol of each primer (see candidate genes), 2x Sybr Green PCR buffer (Bio-Rad, Hercules). The program for QRT-PCR was set to 2 minutes at 50°C, 5 minutes at 95°C, continued by 40 cycles of 95°C for 30 seconds and 30 seconds at 60°C and a melting curve analysis. PCR reactions were performed in the CHROMO4 MJ research PTC200 (Bio-Rad, Hercules). The data was analyzed according to the qgene 96 programme [37].

### Candidate genes

Homologs of several genes that play a prominent role in the proline metabolism pathway were identified in potato by querying the Potato database (http://solgenomics.net/) using Blast searches. We identified unigenes with homology to *P5CS1* (delta1-pyrroline-5-carboxylate synthase), *P5CR* (Delta 1-pyrroline-5-carboxylate reductase), and *PDH* (Proline dehydrogenase) from *Arabidopsis*. Homology to *Arabidopsis* was determined on the amino acid level. Subsequently, transcript levels of unigenes were determined by RT-PCR using cDNA from plants subjected to 0 mM and 60 mM NaCl (data not shown). Those unigenes that responded to salinity were chosen for QRT-PCR analysis and listed in Table 3.

**Table 3:** Selection of genes from the proline metabolism pathway.

Gene	Annotation/Gene accesion	Homology to	Primer 5′ 3′
*P5CS1*	AT2G39800.1	SGN-U271254	Fw. TTAAAGAGGACGGAGCTTGC
			Rv. CAGTGCATCAGGTCGTGACT
*P5CR*	AT5G14800.1	SGN-U280492	Fw. CCAATTCCAGCCGATTCATA
			Rv. GAAGCAGGCAATATCCCAGA
*PRODH*	AT3G30775.1	SGN-U276105	Fw. CCACCGATAATGAATCTTGTGAAC
			Rv. TTGCAGAAGATTCGGGAAGT

Homology in potato was found by performing blast searches of sequences from *Arabidopsis thaliana* to a database containing potato SGN-unigenes (http://solgenomics.net/).
